# Changes in the composition of intestinal fungi and their role in mice with dextran sulfate sodium-induced colitis

**DOI:** 10.1038/srep10416

**Published:** 2015-05-27

**Authors:** Xinyun Qiu, Feng Zhang, Xi Yang, Na Wu, Weiwei Jiang, Xia Li, Xiaoxue Li, Yulan Liu

**Affiliations:** 1 Department of Gastroenterology, Peking University People’s Hospital, Beijing, China; 2 CAS Key Laboratory of Pathogenic Microbiology and Immunology, Institute of Micro-biology, Chinese Academy of Sciences, Beijing, China; 3 Institute of Clinical Molecular Biology & Central Laboratory, Peking University People’s Hospital, Beijing, China

## Abstract

Intestinal fungi are increasingly believed to greatly influence gut health. However, the effects of fungi on intestinal inflammation and on gut bacterial constitution are not clear. Here, based on pyrosequencing method, we reveal that fungal compositions vary in different intestinal segments (ileum, cecum, and colon), prefer different colonization locations (mucosa and feces), and are remarkably changed during intestinal inflammation in dextran sulfate sodium (DSS)-colitis mouse models compare to normal controls: *Penicillium, Wickerhamomyces, Alternaria,* and *Candida* are increased while *Cryptococcus, Phialemonium, Wallemia* and an unidentified Saccharomycetales genus are decreased in the guts of DSS-colitis mice. Fungi-depleted mice exhibited aggravated acute DSS-colitis associated with gain of *Hallella*, *Barnesiella*, *Bacteroides*, *Alistipes*, *and Lactobacillus* and loss of butyrate-producing *Clostridium* XIVa, and *Anaerostipes* compare with normal control. In contrast, bacteria-depleted mice show attenuated acute DSS-colitis. Mice with severely chronic recurrent DSS-colitis show increased plasma (1,3)-β-D-glucan level and fungal translocation into the colonic mucosa, mesenteric lymph nodes and spleen. This work demonstrate the different roles of fungi in acute and chronic recurrent colitis: They are important counterbalance to bacteria in maintaining intestinal micro-ecological homeostasis and health in acutely inflamed intestines, but can harmfully translocate into abnormal sites and could aggravate disease severity in chronic recurrent colitis.

The human gastrointestinal (GI) canal is colonized with 10–100 trillion commensal microbiota. Up to 98% of GI microbiota are bacteria, and the other 2% comprise fungi, viruses, and protists, among others^1^. Because bacteria dominate intestinal microbial communities, most studies have focused on the role of bacteria in tuning mucosal immunity and promoting intestinal health^2^, whereas the functions of other microbes have been neglected.

Fungi are eukaryotic organisms that colonize the guts of many mammals. They can be detected in almost all GI sections by metagenomics^3^,^4^ and interact closely with GI commensal bacteria^3^,^5^. Previous studies identified numerous fungi from human fecal samples. Although the *Candida* genus is most abundant, the *Aspergillus*, *Cryptococcus*, *Mucor*, *Rhodotorula*, and *Trichosporon* genera are also prevalent in humans and are thought to migrate from the respiratory tract or skin^4^,^6^.

GI microbiota can be divided into two distinct ecosystems: the luminal microbiota (mostly present in the feces) and the mucosal microbiota (bound to the mucosa and adhered to the intestinal epithelium)^7^. Although past studies identified the relationships between fungi and intestinal inflammation, most employed classical culture methods. DNA-based studies of fungi are therefore warranted to obtain sufficient information for taxon assignments. Additionally, most studies on human GI microbiota analyzed fecal specimens, but the microbiota therein may not participate directly in disease initiation. The mucosal microbiota, although fewer in number, reflect more microbial signals than fecal microbiota and directly affect the host immune response^8^,^9^. Specific features of mucosa-associated fungal dysbiosis have not been fully characterized, and few studies have made comparisons with luminal controls.

Disturbance of fungal compositions is common in patients with inflammatory bowel disease (IBD) and may aggravate disease condition in compromised host environments^10^,^11^. Recent work demonstrated a significant increase of *Candida albicans* in the guts of patients with Crohn’s disease^12^,^13^, causing delayed mucosal healing and generation of anti-*Saccharomyces cerevisiae* antibodies^4^,^7^,^14^. In dextran sulfate sodium (DSS)-induced murine IBD models^3^,^15^, fungal dysbiosis was characterized by the promotion of opportunistic pathogenic *Candida* and *Trichosporon* and decreased levels of non-pathogenic *Saccharomyces*^3^.

The effect of gut fungi on intestinal inflammation is controversial. A genetic single-nucleotide polymorphism on Dectin-1, an immune cell receptor that recognizes β-glucan on the cell walls of various intestinal fungi, is usually associated with increased severity of ulcerative colitis in patients^3^. Furthermore, Ilieve *et al.*^3^ reported that Dectin-1-deficient mice exhibit more severe experimental colitis accompanied by general expansion of opportunistic gut fungi and fungal invasion into the colonic mucosa. However, Heinsbroek *et al.*^16^ suggested that Dectin-1 deficiency in mice does not affect intestinal inflammation in experimental colitis. Thus, the relationships between fungi and intestinal inflammation require clarification. In addition, the fungal-bacterial interactions in the gut remain an enigma.

Herein, we investigated the fungal distribution in different segments (ileum, cecum, and colon) and locations (mucosa and feces) of the gut in normal and colitis mice, compared the roles of fungi and bacteria in intestinal inflammation, and studied the relationships between them to elucidate the function of fungi in intestinal inflammation and fungal-bacterial interactions in the GI canal. Our findings will be informative for future clinical IBD treatment.

## Results

### Fungal compositions vary among gut segments in normal and acute DSS-colitis mice

**Figure 1:**
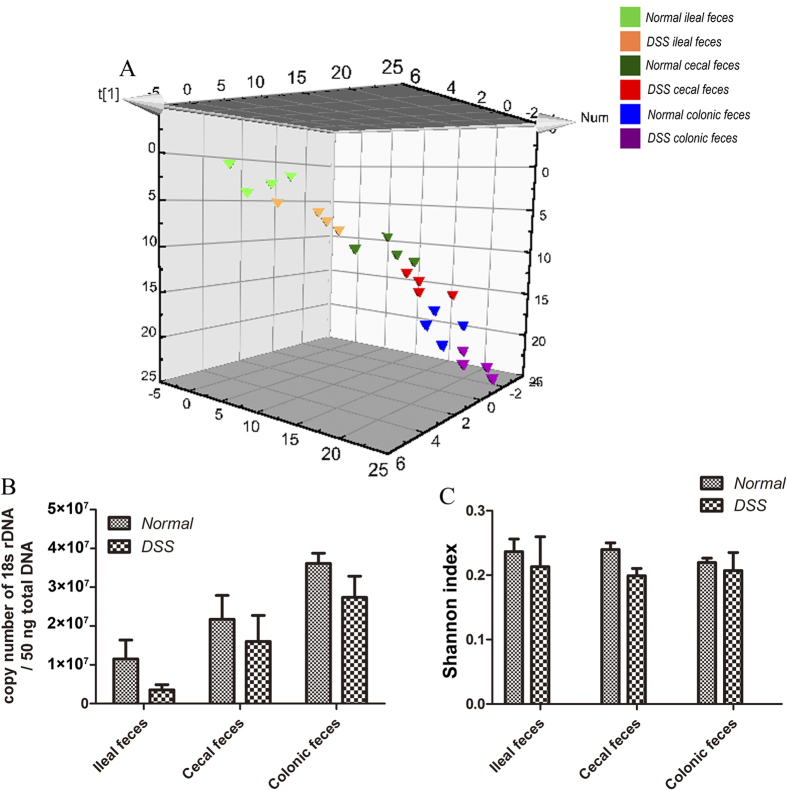
Fungal compositions vary among gut segments in normal and acute DSS-colitis mice. **(A)** Partial least-squares discriminant analysis (PLS-DA) scores plot based on the relative abundance of fungal operational taxonomic units (OTUs) (97% similarity level) in feces from different intestinal segments of normal and acute DSS-colitis mice. **(B)** qRT-PCR of 18S rDNA was performed on 50 ng total DNA isolated from the ileal, cecal, and colonic feces of normal and acute DSS-colitis mice. **(C)** The Shannon-Weiner biodiversity index (Shannon index) was measured to represent the diversity of fungi in the murine fecal samples (n = 4/group).

Although short-term DSS treatment did not significantly injure the small intestinal mucosa (Supplementary Fig. S1A), but the pro-inflammatory cytokines (IL-6, IL-17, and IFN-γ) were increased while the anti-inflammatory cytokine (IL-10) was decreased in the ileum of DSS-treated mice compared to the normal control. (Supplementary Fig. S1C–F). Therefore, we identified the fecal fungi from the ileum, cecum, and colon of normal and DSS-colitis mice (four mice/group) to determine the fungal distributions in different GI segments and their association with normal and inflammatory conditions. We obtained 23,539,240 trimmed sequences (~980,801 sequences/sample) and defined 645 operational taxonomic units (OTUs) using a 97% similarity cut-off value (Supplementary Fig. S2A) to profile the overall structural changes of gut fungi as partial least-squares discriminant analysis (PLS-DA) plots and heat maps. Fungal compositions differed in different intestinal segments and varied between the normal and DSS-colitis groups. (Fig. 1A) The total fungal number showed an increased trend from the ileum to the colon in both normal and DSS-colitis mice (Fig. 1B). The fungal Shannon biodiversity index of the DSS-colitis group was slightly lower than that of normal controls in each gut segment (Fig. 1C).

### Fungal compositions differ in colonic mucosa and feces and change during inflammation

**Figure 2:**
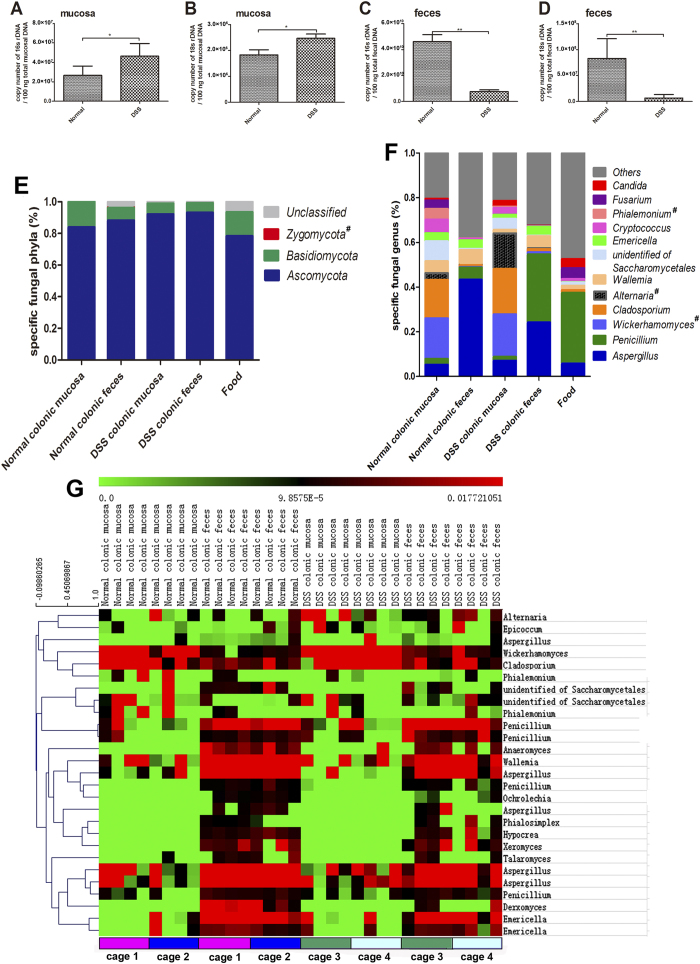
Fungal compositions differ between colonic mucosa and feces and change during intestinal inflammation. Colonic mucosa and feces were collected after the mice were sacrificed. **(A)** 16S rDNA (representing bacteria) and **(B)** 18S rDNA (representing fungi) levels in the colonic mucosa were analyzed by qRT-PCR, and their copy numbers were calculated from the total amount of mucosal DNA (100 ng). **(C)** 16S rDNA and **(D)** 18S rDNA levels in the colonic fecal samples were analyzed by qRT-PCR, and their copy numbers were calculated from the total amount of fecal DNA (100 ng). The values are expressed as mean ± SEM. *P < 0.05, **P < 0.01, ***P < 0.001. **(E)** Phyla and **(F)** genera changes in 12 major fungal genera (average relative abundance **≥**0.01 on average in colonic samples) for colonic mucosal and fecal samples after DSS treatment. Fungal compositions in the mouse diet were also detected. The *Y*-axis indicates the relative abundance of fungi at the phylum or genus level. Fungi found in the colon but not the food are marked with an octothorpe (#). **(G)** Heat maps showing the 27 core OTUs of fungal communities inferred from mucosal and fecal ITS1-2 sequences, with each mouse shown individually. The eight mice in each of the two treatment groups were each housed in two cages. The distribution of mice in cages is indicated at the bottom of the columns. The colored squares in each row indicate the relative abundance of the OTU among the 32 subjects. (n = 8/group). *P < 0.05, **P < 0.01, ***P < 0.001.

18S rDNA and 16S rDNA primer-amplified fragments were used to quantitatively analyze the fungal and bacterial contents in the gut, respectively^17^. The 18S rDNA (fungal) content was significantly increased in the colonic mucosa but decreased in the colonic feces of DSS-colitis mice, exhibiting the same trend as 16S rDNA (bacterial) (Fig. 2A–D). To analyze the colonic fungal diversity, 38,336,293 trimmed ITS1-2 sequences were obtained and further analyzed (~1,127,538 sequences/sample). Three phyla (Ascomycota, Basidiomycota, and Zygomycota) were identified in the colons of both groups but in different proportions: Ascomycota was significantly higher while Basidiomycota was lower in the colons of DSS-colitis mice (Fig. 2E and Supplementary Fig. S3). Moreover, Zygomycota was absent in the mouse diet. Twelve major genera (average relative abundance ≥0.01 in colonic samples) belonging to eight families were identified: *Aspergillus*, *Penicillium*, *Cladosporium*, *Wickerhamomyces*, *Alternaria*, *Wallemia*, *Emericella*, *Cryptococcus*, *Phialemonium*, *Fusarium*, *Candida*, and an unidentified Saccharomycetales genus (Fig. 2F). Interestingly, *Aspergillus* and *Penicillium* were closely associated with the lumina (feces), while *Cladosporium*, *Wickerhamomyces*, *Alternaria*, *Cryptococcus*, *Phialemonium*, *Candida*, and the unidentified Saccharomycetales genus were associated with the mucosa (Supplementary Fig. S4A–I). Additionally, *Wallemia*, *Emericella*, and *Fusarium* did not display obvious preferences for colonization in the mucosa or the enteric cavity (Supplementary Fig. S4J–L). Fungal compositions also differed between the normal and DSS groups: *Cryptococcus*, *Phialemonium*, *Wallemia* and the unidentified Saccharomycetales genus were decreased in the inflamed gut (Fig. 2F and Supplementary Fig. S4F-H and 4J), while *Penicillium, Wickerhamomyces, Alternaria,* and *Candida* were increased (Fig. 2F and Supplementary Fig. S4B, 4D, 4E, and 4I). However, only the proportions of fecal *Candida* and *Wickerhamomyces* showed statistically significant differences between the two groups, probably owing to the limited sample size. Notably, nine of the 12 major genera were present in the mouse diet (Fig. 2F).

We defined 27 OTUs in more than 50% of the mucosal and fecal samples as the core microbiome, according to a method described previously^18^. PLS-DA score plots (Supplementary Fig. S5) and heat maps (Fig. 2G) were constructed for the core OTUs and show that fungal communities differ between the normal and colitis groups but show greater similarity within a location (even between groups) than between locations in the same group. Also, the fungal composition of mice with the same treatment (but in different cages) are similar to each other for both mucosal and fecal specimens. (Fig. 2G and Supplementary Fig. S5)

### Fungal translocation occurs in mice with severely chronic recurrent colitis

**Figure 3:**
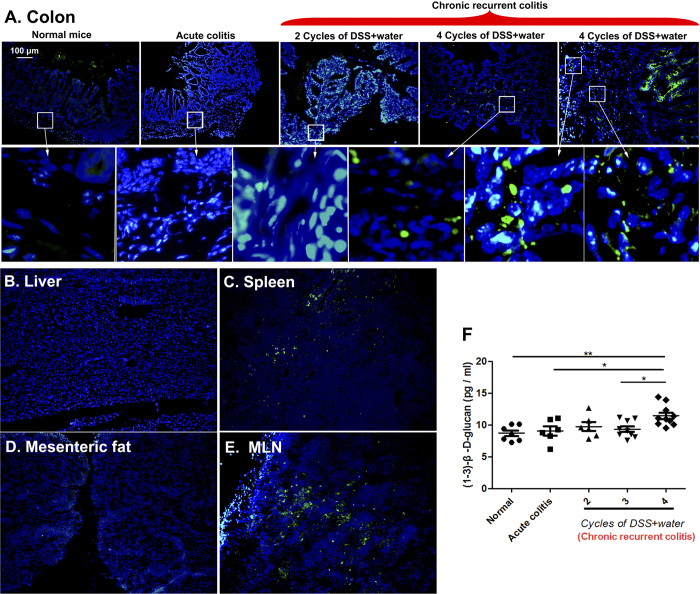
Fungal translocation in mice with chronic recurrent colitis. **(A)** Colon sections from normal mice, acute colitis mice, and mice treated with two and four cycles of DSS + water were stained with anti-fungal antibody (green) and counterstained with DAPI (blue). **(B–E)** Liver, spleen, mesenteric fat, and mesenteric lymph node (MLN) sections from mice exposed to four cycles of DSS + water were stained with anti-fungal antibody (green) and counterstained with DAPI (blue). **(F)** (1,3)-β-D-glucan levels were detected in murine plasma by enzyme-linked immunosorbent assay.

Mice exposed to four cycles of DSS + water exhibited the most severe inflammation, as determined by detection of mucosal pro-inflammatory cytokines (IL-17A, IL-23, TNF-α, and IFN-γ) and histological assessment (Supplementary Fig. S6A–F). Fungi invaded the colonic mucosa and translocated from the intestinal lumen into some extra-enteric organs (spleen and mesenteric lymph node [MLN]) in mice treated with four cycles of DSS + water (Fig. 3A–E). The fungal 18S rDNA levels in spleen and MLN of mice treated with four cycles of DSS + water were significantly increased compared to the normal control. (Supplementary Fig. S7) However, no significant fungal translocation was found in the intestinal walls of normal mice, acute colitis mice, or mice treated with two cycles of DSS + water, as determined by the immunofluorescent assay (Fig. 3A and Supplementary Fig. S8). The plasma concentration of (1,3)-β-D-glucan in mice with chronic recurrent colitis was higher than that in normal mice, and a significant increase in this index was found in mice treated with four cycles of DSS + water relative to normal controls (Fig. 3F).

### Occludin and ZO-1 are decreased in the colons of mice with chronic recurrent colitis

Quantitative real-time PCR revealed that two key tight-junction proteins (occludin and ZO-1) were decreased in the colons of chronic colitis mice and were significantly decreased in mice treated with four cycles of DSS + water (Supplementary Fig. S9A). Immunofluorescence localization of occludin and ZO-1 in the mouse distal colon showed morphological changes similar to those observed for colon mRNA levels (Supplementary Fig. S9B).

### Fungal depletion aggravates acute colitis in mice

**Figure 4:**
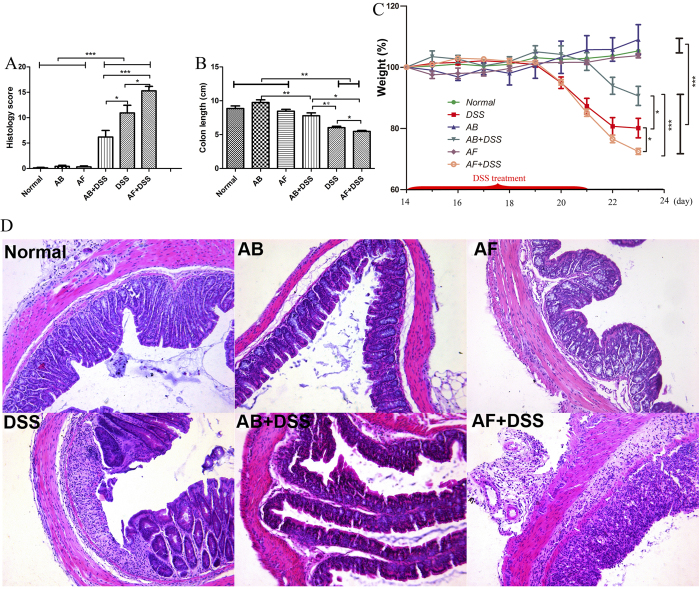
Fluconazole (anti-fungal, AF) treatment exacerbates acute DSS-colitis in mice. Six groups of mice were treated as shown in Table 2. **(A)** Histology scores and **(B)** colon lengths were measured on day 23 after sacrifice. **(C)** Weights were measured on days 14–23, and the percent weight change was calculated. The values are expressed as mean ± SEM. **(D)** Hematoxylin and eosin staining of representative cross-sections of murine distal colon (HE, ×200). Colons of normal, antibiotic cocktail (AB), and AF mice have normal appearances. AB + DSS mice exhibit slight inflammation with a low level of lymphocyte infiltration. DSS mice display mucosal and submucosal inflammation, bowel wall thickening, and a moderate level of lymphocyte infiltration and regeneration with crypt depletion. AF + DSS mice show transmural inflammation, extensive lymphocyte infiltration, and loss of the entire crypt and epithelium. (n = 8–12/group). *P < 0.05, **P < 0.01, ***P < 0.001.

**Figure 5:**
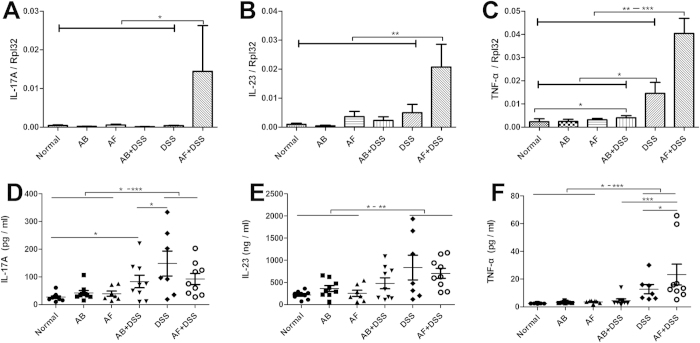
Inflammatory cytokine (IL-17A, IL-23, and TNF-α) levels in the colonic mucosa and serum . **(A–C)** mRNA expression levels of IL-17A, IL-23, and TNF-α in the colon were measured by qRT-PCR and normalized to Rpl32 mRNA. (n = 8–12/group). **(D–F)** IL-17A, IL-23, and TNF-α cytokine serum concentrations were measured by MILLIPLEX^TM^ Immunoassays. The values are expressed as mean ± SEM. *P < 0.05, **P < 0.01, ***P < 0.001.

The fluconazole (AF) + DSS group showed the greatest weight loss, shortest colon length, and highest histological score among the six groups, followed by the DSS and antibiotic cocktail (AB) + DSS groups; there were no obvious differences among the normal, AB, and AF groups (Fig. 4A–D). AF + DSS and DSS mice showed higher levels of IL-17A, IL-23, and TNF-α in colonic mucosa and serum samples than the normal, AB, AF, and AB + DSS groups (Fig. 5A–F). Notably, the AF + DSS group revealed the highest concentration of TNF-α, followed by the DSS group (Fig. 5F).

### Fungal depletion substantially changes the compositions of colonic mucosal bacteria

**Figure 6:**
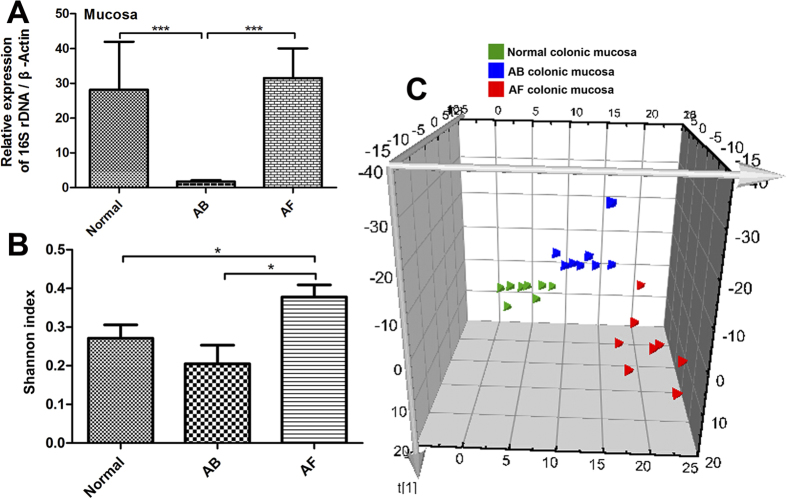
Change in mucosal bacterial compositions by antibiotic cocktail (AB) and fluconazole (AF) treatment. **(A)** The 16s rDNA levels in the colonic mucosa of AB mice were significantly decreased relative to normal and AF mice. **(B)** The bacterial Shannon index was significantly increased in the mucosa of AF mice relative to normal and AB mice. **(C)** Partial least-squares discriminant analysis (PLS-DA) scores plot based on the relative abundance of bacterial OTUs (97% similarity level) in the colonic mucosa of normal, AB, and AF mice. *P < 0.05, **P < 0.01, ***P < 0.001.

**Figure 7:**
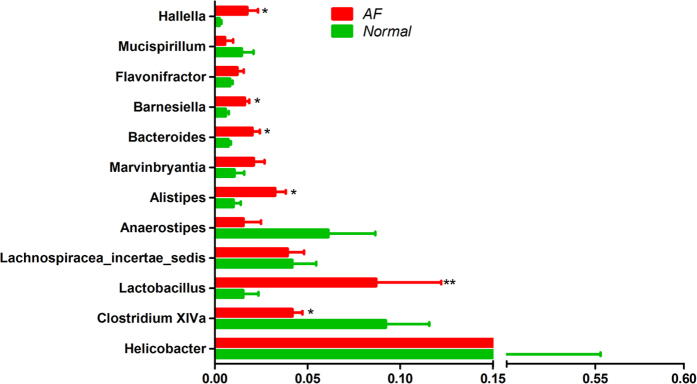
Comparison of 12 main bacterial genera (relative abundance ≧0.01 on average) in the colonic mucosa of normal and AF mice. *P < 0.05, **P < 0.01.

16S rDNA levels were sharply reduced in the AB mucosa (93.7% decrease) but remained unchanged in the AF mucosa compared to normal controls (Fig. 6A). To analyze the bacterial diversity in the colon, we obtained 24,152,385 reads of the 16S rDNA V3 region (~1,006,349 reads/sample) (Supplementary Figure S2B). Interestingly, the bacterial diversity of the AF group was increased relative to the normal control, while that of the AB group showed a decreased trend (Fig. 6B). We further compared the genera of mucosal bacterial populations in AF and normal mice and found that AF mice had increased relative abundances of *Hallella*, *Barnesiella*, *Bacteroides*, *Alistipes*, and *Lactobacillus* but decreased relative abundances of *Clostridium* XIVa and *Anaerostipes* (Fig. 7).

## Discussion

Fungi have been associated with several gastrointestinal diseases^19^, but data on relationships between intestinal fungi and intestinal inflammation are limited. Previous studies have reported a substantial shift of gut fungi in paediatric and adult patients with IBD^20^,^21^, suggesting an important and underappreciated role of fungi on the genesis and maintenance of IBD. Herein, we analyzed the fecal fungi in different GI segments with high-throughput sequencing and bioinformatics and demonstrated that the fungal compositions varied among GI segments and that fungal relative abundance showed an increased trend from ileum to colon. Moreover, the fungal burden showed dichotomous behavior during intestinal inflammation: it increased in the mucosa but decreased in the feces, similar to observations for intestinal bacteria.

Previous studies on the fungal compositions of the GI canal mostly analyzed fecal samples; however, a large gap remains in our knowledge of the overall characteristics of intestinal fungi (both mucosal and fecal). Because the colonic segment is colonized with the most abundant fungi^3^,^6^ and shows severe inflammation in IBD patients and DSS-colitis mice, we investigated the fungal compositions in both locations (mucosa and feces) of the colon from normal and colitis mice. Although fungi in the intestine favored different colonization locations, the relative abundance of several mucosal and fecal fungi still followed the same trend during colitis. *Cryptococcus*, *Wallemia*, and an unidentified Saccharomycetales genus were less abundant in the inflamed gut, whereas *Wickerhamomyces, Alternaria*, and *Candida* increased. Iliev *et al.*^3^ reported that the proportions of opportunistic pathogenic *Candida* and *Trichosporon* increased, whereas that of non-pathogenic *Saccharomyces* was suppressed in the feces of DSS-colitis mice. Notably, *Candida*, the major genus in the murine gut^3^, did not constitute a high proportion of fungi in our study. Hence, we detected the fungal compositions in the diet and identified nine of the 12 major intestinal fungal genera. The two most abundant genera (*Aspergillus* and *Penicillium*) in the diet account for a considerable proportion of fungi in the mouse gut, emphasizing the important role of diet in shaping intestinal fungal compositions. Dollive *et al.*^17^ reported that intestinal commensal fungal communities are more variable than those of bacteria, and can differ among individuals in different cages but within a treatment group. However, the differences in fungal composition between cages in our study were not significant, as shown by the heat map (Fig. 2G) of fungal composition for mucosal and fecal specimens. Moreover, the PLS-DA (Supplementary Fig. S5) based on the core OUTs (OTUs present in 50% or more of the mucosal and fecal samples) indicates that fungal communities in mice with the same treatment (between cages) have greater tendency to cluster together. Experimental differences in environmental facility, diet, and even the treatment time (nine days herein, consisting of seven days of water containing 2.5% DSS followed by two days of distilled water, compared to 76 days in the previous study) between these studies may account for the different observations.

Several previous studies documented that intestinal bacteria can translocate into extra-enteric organs and aggravate inflammation in patients with Crohn’s disease and in experimental colitis models^22^,^23^,^24^,^25^. However, whether fungal translocation occurs in colitis models was not fully determined. Our results show that fungi can invade the colonic mucosa and translocate into the spleen and MLN in severe colitis mice but not in normal mice, acute colitis mice, or mice with less severe chronic colitis. Remarkably, two key tight-junction proteins, occludin and ZO-1^26^, are decreased in the colons of severe colitis mice, reflecting the probable involvement of damaged gut mucosal barriers in permitting intestinal microbial (fungi, bacterial, and other microrganisms) translocation^27^,^28^. Although we cultured the fungi from the spleen and MLN, few fungi grew (data not shown); however, the 18S rDNA level was significantly increased in the spleen and MLN of mice with chronic recurrent colitis compared with the normal control. Interestingly, the bacteria translocated into these extra-enteric organs can be easily cultured (data not shown). It is thus unclear whether the translocated fungi were viable after migration into the spleen and MLN of colitis mice. Further studies are needed to clarify which fungi translocate and to evaluate their effects in these organs. Moreover, elevated concentrations of plasma (1,3)-β-D-glucan, an important marker of invasive fungal infection^29^,^30^, were found in mice with chronic recurrent colitis, which complements a previous clinical study that detected plasma (1,3)-β-D-glucan in IBD patients^29^, suggesting that fungi could migrate into the blood during chronic intestinal inflammation.

IBD patients and animal models commonly have an abundance of gut bacteria in the inflamed mucosa, and antibiotics effectively attenuate intestinal inflammation^31^,^32^. We tested whether colitis can be mitigated by eliminating gut fungi, which also gather in the inflamed mucosa, by evaluating the severity of acute DSS-colitis in bacteria-depleted and fungi-depleted mice. Unexpectedly, fungi-depleted mice exhibited aggravated colitis manifested by increased weight loss, histological score, and proinflammatory cytokine production and decreased colon length. However, intestinal inflammation was alleviated in bacteria-depleted mice, consistent with previous studies^32^. Therefore, we hypothesized that bacteria are the major driving force in acute enteric inflammation.

Previous studies reported more obvious microbial dysbiosis in the mucosa of IBD patients relative to the feces^9^,^33^, while studies of mucosal microbiota are relatively less than that of fecal ones. To clarify whether fungal elimination violated the mutualism of bacterial flora in the gut, thus exacerbating colitis, we identified the bacterial distributions in the colonic mucosa of normal, AB, and AF mice. We found that the 23-day antibiotic cocktail treatment exhibited a decreased trend in affecting the mucosal bacterial diversity and cleared almost all gut bacteria; whereas long-term use of fluconazole prominently augmented the mucosal bacterial diversity in the gut without affecting the total bacterial number. We compared the bacterial compositions of the normal and AF groups and found increased relative abundances of *Hallella*, *Barnesiella*, *Bacteroides*, *Alistipes*, and *Lactobacillus* but decreased abundances of *Clostridium* XIVa and *Anaerostipes* in AF mice. No previous studies, to our knowledge, have reported the relationships between *Hallella*, *Barnesiella*, and *Alistipes* and intestinal inflammation. Li *et al.*^34^ observed a higher relative abundance of oral *Hallella* in periodontitis patients, and Baxter *et al.*^35^ reported a strong positive correlation between intestinal *Alistipes* abundance and the colonic tumor burden. Additionally, levels of *Bacteroides* usually increase in patients with IBD or colorectal carcinoma^35^,^36^. However, *Clostridium* XIVa and *Anaerostipes* are believed to improve colonic health by producing butyrate^36^,^37^. Thus, the loss of intestinal fungi may trigger proliferation of pathogenic microbiota while reducing the proportion of beneficial butyrate-producing bacterial taxa, thereby exacerbating intestinal inflammation. Notably, *Lactobacillus*, a probiotic claimed to benefit patients with IBD^2^, was increased in AF mice. Because gut inflammation is complexly regulated by the overall intestinal composition, the ability of *Lactobacillus* to counterbalance deterioration of the gut micro-ecological environment remains to be studied. Moreover, we found that *Helicobacter spp*. is the most abundant genus in the mouse colon, and several *Helicobacter* species (e.g., *H. hepaticus*, *H. bilis,* and *H. muridarum, etc.*) were previously reported to be colitogenic^38^. Thus, whether the high abundance of *Helicobacter* present in the murine gut affected the total intestinal microbial homeostasis and the murine phenotype in the DSS treatment require further investigation.

Herein, we provide novel insights into the distribution of fungi in the murine gut, compare the fungal compositions between mucosal and fecal samples in mice with or without intestinal inflammation, and reveal the roles of fungi in acute and chronic recurrent colitis. Fungi are an important counterbalance to bacteria in maintaining intestinal micro-ecological homeostasis and health in the case of acute colitis but may aggravate the severity of chronic recurrent colitis. Our results suggest that fungal depletion may not attenuate acute colitis in patients with IBD, but this conclusion requires clinical confirmation. However, a previous study reported that pathogenic fungi more frequently colonize the colonic mucosa in patients with chronic ulcerative colitis and that anti-fungal treatment accelerates remission of clinical symptoms in these patients^7^. Hence, future investigation of the roles of fungi in chronic IBD is warranted. Further studies are also required to define the fungal compositions in detail, identify the probiotic and pathogenic fungi in the gut, uncover the taxonomic level of intestinal fungal and bacterial interactions, and specify the mechanism underlying the regulation of intestinal health by enteric microbiota.

## Materials and Methods

### Animals and experimental colitis model

Six-week-old C57B/L6J mice were purchased from the Experimental Animal Center, Academy of Military Medical Sciences (Beijing, China). Four to five Mice were maintained in a single cage and raised under specific pathogen-free conditions at the Animal Center of Peking University People’s Hospital and fed standard rodent food and distilled water for four weeks before beginning experiments. To induce acute colitis, mice were given drinking water containing 2.5% (w/v) DSS (MP Biomedicals, Aurora, OH) ad libitum for seven days and distilled water for two additional days before sacrifice. To induce chronic recurrent colitis, mice were treated with two to four 14-day cycles consisting of seven days of water containing 2.5% DSS followed by seven days of distilled water (DSS + water), a protocol modified from Batra *et al.*^39^, before sacrifice. All animal experiments were approved by the Committee for Laboratory Animal Management of Peking University and treated humanely in accordance with the National Institutes of Health guidelines on the ethical use of animals.

### DNA isolation

Immediately after sacrifice, intestinal fecal samples from the ileum, cecum, and colon were collected, frozen in liquid nitrogen, and stored at −80 °C until further processing. The colons were then opened longitudinally and briefly washed three times in sterile phosphate-buffered saline (PBS) to remove unattached or loosely attached microbiota. DNA in fecal and colonic tissue samples (refered to as mucosa in our study) was extracted using a FastDNA® SPIN Kit for Feces (MP Biomedicals) according to the manufacturer’s instructions and quantified on a NanoDrop 1000 spectrophotometer (Thermo Scientific).

### Library construction

We amplified internal transcribed spacer regions 1 and 2 (ITS1-2, representing fungi) and 16S rDNA (representing bacteria) with the primers listed in Supplementary Table S1. Primer sets were modified with Illumina adapter regions for sequencing on the Illumina GAIIx platform, and reverse primers were modified with an 8-bp Hamming error-correcting barcode to distinguish among samples. The DNA template (100 ng) was combined with 5 μL PCR buffer, 1 μL dNTPs, 0.25 μL HotStarTaq® Plus DNA Polymerase (Qiagen), and 2.5 pmol of each primer in 50 μL total volume. Reactions consisted of an initial step at 95 °C for 5 min; 25 (16S rDNA) or 38 cycles (ITS1-2 rDNA) of 94 °C for 45 s, 55 °C for 45 s, and 72 °C for 60 s; and a final extension at 72 °C for 10 min. DNA products were checked by 1.5% (w/v) agarose gel electrophoresis in 0.5 mg/mL ethidium bromide and purified with the Qiaquick gel extraction kit (Qiagen).

### 16S and 18S rDNA gene quantitative analysis

Primers for 16S and 18S rDNA (Supplementary Table S1) were used to quantitatively analyze the concentrations of bacteria and fungi in the gut, respectively. PCR reactions consisting of 10 μL SYBR Green PCR master mix (TOYOBO), 1.6 μL (10 nM) each primer, 100 ng template cDNA, and 7.4 μL water in a total volume of 20 μL were carried out on an ABI StepOne Plus Sequence Detection System (Applied Biosystems), and thermocycling reactions consisted of 1 min at 95 °C followed by 40 cycles of 15 s at 95 °C, 15 s at 56 °C, and 45 s at 72 °C. Melting curve analysis demonstrated a single amplicon for each reaction. To detect the contents of bacteria and fungi in the mucosal and fecal samples, the amounts of 16S rDNA and 18S rDNA were calculated using standard curves constructed with known concentrations of plasmid DNA (Transgene, Beijing, China) containing the respective amplicons.

### Bioinformatic analysis

Sequences of ITS1-2 and the V3 region of 16S rDNA in mouse intestine and food were detected using an Illumina HiSeq 2000 platform (reconstructed cDNA sequence: 2 × 150 bp). Ribosomal Database Project (RDP) Classifier 2.8 was used for taxonomical assignment of all sequences at 50% confidence after the raw sequences were identified by their unique barcodes. OTUs present in 50% or more of the gut (mucosal and fecal) samples were identified as core OTUs^18^. PLS-DA of core OTUs was performed using Simca-P version 12 (Umetrics), and a heat map was generated with Multi-Experiment Viewer (*MeV)* software to visualize and cluster the fungal community into different groups. Community diversity was measured by the Shannon-Weiner biodiversity index (Shannon index)^40^.

### Fungal translocation in mice with chronic recurrent colitis

We stained for fungal translocation using FITC-conjugated rabbit anti-*Candida* polyclonal antibody (Meridian Life Science, Cincinnati, OH), which can cross-react with a wide variety of fungal species but not bacteria, as an “anti-fungal antibody.”^3^ Frozen sections (6 μm) of the liver, spleen, MLN, mesenteric fat, and distal colon from normal mice and mice with acute or chronic recurrent colitis were fixed in 4% paraformaldehyde for 10 min, rinsed twice with PBS for 10 min, and incubated with 10 μL of goat serum working solution (Beijing Zhongshan Golden Bridge Biotechnology, Beijing, China) at room temperature for one hour. The fluorescent anti-fungal antibody was added at 4 °C overnight. Finally, slides were rinsed with PBS, stained with an anti-fade reagent with DAPI (Invitrogen), and examined using a Leica Observer Fluorescence microscope. Colons and extra-enteric organs from AF mice were used in control staining. 18S rRNA levels in extra-enteric organs were determined using quantitative reverse-transcription polymerase chain reaction (qRT-PCR) normalized to glyceraldehyde -3-phosphate dehydrogenase (GAPDH)^41^. The concentration of (1,3)-β-D-glucan in murine plasma was determined by the detection reagent kit (Associates of Cape Cod).

### Immunofluorescence analysis of occludin and ZO-1

Frozen sections (8 μm) of distal colon from normal and DSS-induced chronic colitis mice were fixed in 4% paraformaldehyde, rinsed in PBS, and incubated in goat serum working solution containing 0.1% Triton-X for one hour. The primary antibody, polyclonal rabbit anti-ZO-1 (1.5 μg/mL, Abcam, Cambridge, MA) or rabbit anti-occludin (1.5 μg/mL, Abcam) was added overnight at 4 °C. After three 20-min washes in PBS, the secondary antibody, Alexa Flour® 488 goat anti-rabbit IgG (1:800, Invitrogen), was added for one hour. Slides were washed three times for 30 min in PBS and mounted in DAPI (Invitrogen).

### Comparison of fungal and bacterial roles in DSS-induced colitis

Mice were randomly divided into six groups: normal, DSS, antibiotic cocktail (AB), fluconazole (anti-fungal, AF), AB + DSS, and AF + DSS. To deplete intestinal bacteria in the AB and AB + DSS groups, mice were given an antibiotic cocktail containing 1 g/L ampicillin (Sigma, St. Louis, MO), 500 mg/L vancomycin (Sigma), 1 g/L neomycin sulfate (Sigma), and 1 g/L metronidazole (Sigma)^3^ in drinking water on days 1–23; to deplete fungi in the AF and AF + DSS groups, mice were given 0.5 mg/mL fluconazole (Sigma)^3^ in drinking water on days 1–23. In the DSS, AB + DSS, and AF + DSS groups, 2.5% (w/v) DSS was added to drinking water only on days 15–21 (Supplementary Table S2).

### Inflammation scoring and body weight

Mice in all groups were weighed on days 14–23. After sacrifice on day 23, colon tissues (2 cm above the anal canal) were treated and stained with hematoxylin and eosin as previously described^42^. Inflammation was graded by two independent blinded observers according to an established grading system^43^.

### Quantitative reverse-transcription polymerase chain reaction (qRT-PCR)

To detect the levels of inflammatory cytokines (IL-6, IL-10, IL-17A, IL-23, IFN-γ, and TNF-α) and tight-junction proteins (occludin and ZO-1) in the colonic (or ileal) mucosa, total RNA was extracted from the proximal and distal colon (or ileum) by the TRIzol method (Gibco) and converted into cDNA^42^. Rpl32, a stable gene during gut inflammation, was used as the internal control^3^. Primers are listed in Supplementary Table S3. All samples were analyzed in duplicate in a single 96-well reaction plate, and data were analyzed according to the 2-∆CT method^3^.

### **MILLIPLEX** ^**TM**^ immunoassays

Blood was collected, coagulated, centrifuged (10 min at 3000 × *g*), and stored at −80 °C until further processing. The concentrations of serum inflammatory cytokines (IL-17A, IL-23, and TNF-α) were determined using magnetic bead-based multiplex assays (BioRad) according to the manufacturer’s instructions.

### Statistical analysis

Data are expressed as mean ± SEM. Statistical analysis was performed with Statistical Package for Social Science version 17.0 software (SPSS Inc., Chicago, IL). ANOVA and Mann–Whitney U-tests were used for analysis. Statistical significance was determined for *P* < 0.05.

## Additional Information

**How to cite this article**: Qiu, X. *et al.* Changes in the composition of intestinal fungi and their role in mice with dextran sulfate sodium-induced colitis. *Sci. Rep.* **5**, 10416; doi: 10.1038/srep10416 (2015).

## Supplementary Material

Supporting Information Supplementary Figures 1-3 and Supplementary Tables 1-3

Supplemetary Dataset
