# Supplementary material for: Changes in the composition of intestinal fungi and their role in mice with dextran sulfate sodium-induced colitis
Source: Sci Rep. 2015 May 27;5:10416. doi: 10.1038/srep10416 (PMC4445066; doi:10.1038/srep10416)
Supplement: Supporting Information — Supplementary Figures 1-3 and Supplementary Tables 1-3 [file srep10416-s1.pdf]

Supplementary Information

Changes in the composition of intestinal fungi and their role in mice with dextran sulfate sodium-induced colitis

Xinyun Qiu[1], Feng Zhang[1], Xi Yang[2], Na Wu[3], Weiwei Jiang[1], Xia Li[1], Xiaoxue Li[1], Yulan Liu[1]*

[1]Department of Gastroenterology, Peking University People's Hospital, Beijing, China.

[2] CAS Key Laboratory of Pathogenic Microbiology and Immunology, Institute of Micro-biology, Chinese Academy of Sciences, Beijing, China.

[3] Institute of Clinical Molecular Biology & Central Laboratory, Peking University People's Hospital, Beijing, China.

*Corresponding author.

Mailing address for Yulan Liu:

Department of Gastroenterology, Peking University People's Hospital

No. 11 Xizhimen South Street, Xicheng District, Beijing 100044, China.

Phone: (8610)8832-5559; Fax: (8610)6831-8386;

E-mail: liuyulan@pkuph.edu.cn

Supplementary information includes:
Supplementary Tables S1 to S3, Supplementary Figures S1 to S9.

Supplementary Tables

Supplementary Table S1 Primers for detection of intestinal fungi (ITS1-2 and 18S rDNA) and bacteria (16S rDNA). β-actin was used as an internal standard.

Amplicon	Forward primer (5´–3´)	Reverse primer (5´–3´)
ITS1-2	CTTGGTCATTTAGAGGAAGTAA	GCTGCGTTCTTCATCGATGC
16S (341F&534R)	ATTACCGCGGCTGCTGG	ATTACCGCGGCTGCTGG
18S	ATTGGAGGGCAAGTCTGGTG	CCGATCCCTAGTCGGCATAG
β-actin	ATGACCCAGATCATGTTTGA	TACGACCAGAGGCATACAG

Supplementary Table S2 Experimental treatment groups

group	Day 1- Day 14	Day 15 - Day 21	Day 22 - Day 23
Normal	normal water	normal water	normal water
DSS	normal water	normal water containing 2.5% DSS	normal water
AB	AB water	AB water	AB water
AF	AF water	AF water	AF water
AB+DSS	AB water	AB water containing 2.5% DSS	AB water
AF+DSS	AF water	AF water containing 2.5% DSS	AF water

Mice were treated with different drinking water regimens ad libitum (see Materials and Methods). All mice were sacrificed on day 23. Mucosal and fecal samples were collected from the colon and detected. [a]AB water: water containing 1 g/L ampicillin, 500 mg/L vancomycin, 1 g/L neomycin sulfate, and 1 g/L and metronidazole. [b]AF water: water containing 0.5 mg/mL fluconazole.

Supplementary Table S3 Primers used for the inflammatory cytokines (IL-17A, IL-23, IFN-γ and TNF-α) and tight junction proteins (Occludin and ZO-1) detecting. Rpl32 was used as the housekeeping gene.

Amplicon	Forward primer	Reverse primer
IL-17A	CAGGACGCGCAAACATGA	GCAACAGCATCAGAGACACAGAT
IL-23	TGGCATCGAGAAACTGTGAGA	TCAGTTCGTATTGGTAGTCCTGTTA
IFN-γ	GGATGCATTCATGAGTATTGC	GCTTCCTGAGGCTGGATTC
TNF-α	TCCAGGCGGTGCCTATGT	CACCCCGAAGTTCAGTAGACAGA
IL-6	TGATGCACTTGCAGAAAACA	ACCAGAGGAAATTTTCAATAGGC

IL-10	TACAGCCGGGAAGACAATAA	AAGGAGTCGGTTAGCAGTAT
Occludin	GCTTATCTTGGGAGCCTGGACA	GTCATTGCTTGGTGCATAATGATTG
ZO-1	AGGACACCAAAGCATGTGAG	GGCATTCCTGCTGGTTACA
GAPDH	TCAACAGCAACTCCCACTCTTCCA	ACCCTGTTGCTGTAGCCGTATTCA
Rpl32	AAGCGAAACTGGCGGAAAC	TAACCGATGTTGGGCATCAG

Supplementary Figures

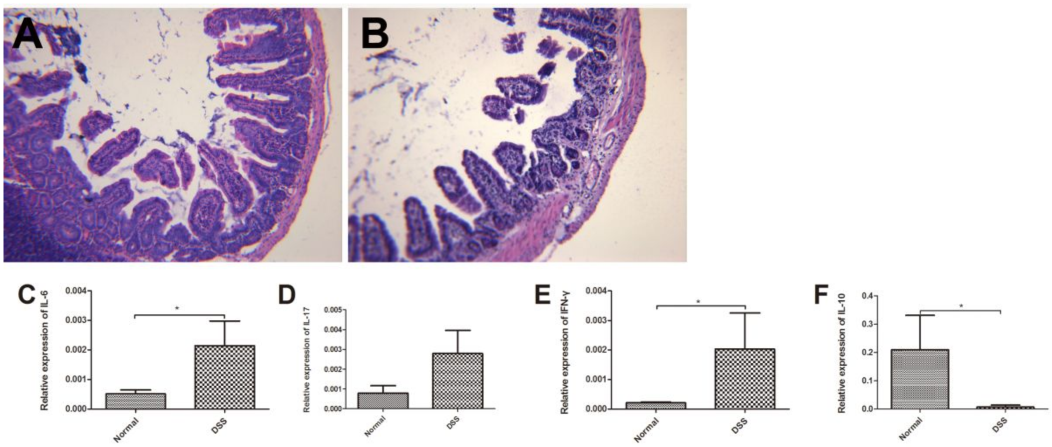

Supplementary Figure S1. Ileum from DSS-treated mice exhibits inflammation. DSS-treated mice were exposed to seven days of water containing 2.5% DSS followed by two days of distilled water. (**A** and **B**) Hematoxylin and eosin staining of distal ileum from normal and DSS-colitis mice (HE, ×200). (**C**-**F**) Increased pro-inflammatory cytokines (IL-6, IL-17 and IFN-γ) and decreased anti-inflammatory cytokine (IL-10) mRNA level were found in the ileum of DSS-treated mice compared to the normal control. (n=6 / group) *$P < 0.05$.

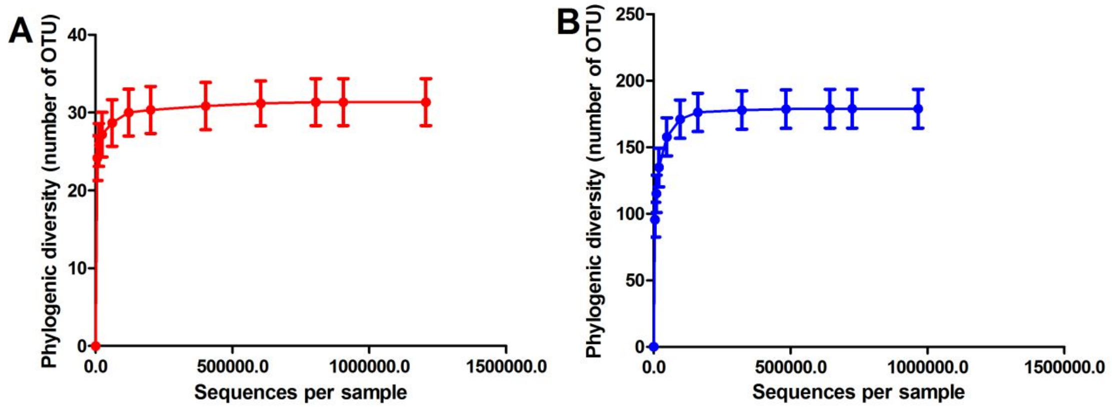

Supplementary Figure S2. Rarefaction analysis of sampling by observed fungal (A) and bacterial (B) OTU method.

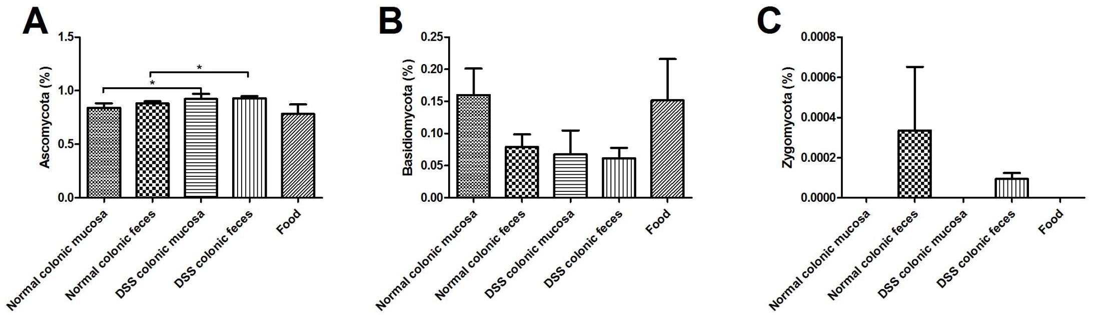

Supplementary Figure S3. Identification of the fungal microbiome at the phylum level in the mouse food and in mucosal and fecal samples from mice with or without acute DSS-induced colitis. Samples from mice: n = 8; Samples from mouse food: n = 2. *$P < 0.05$.

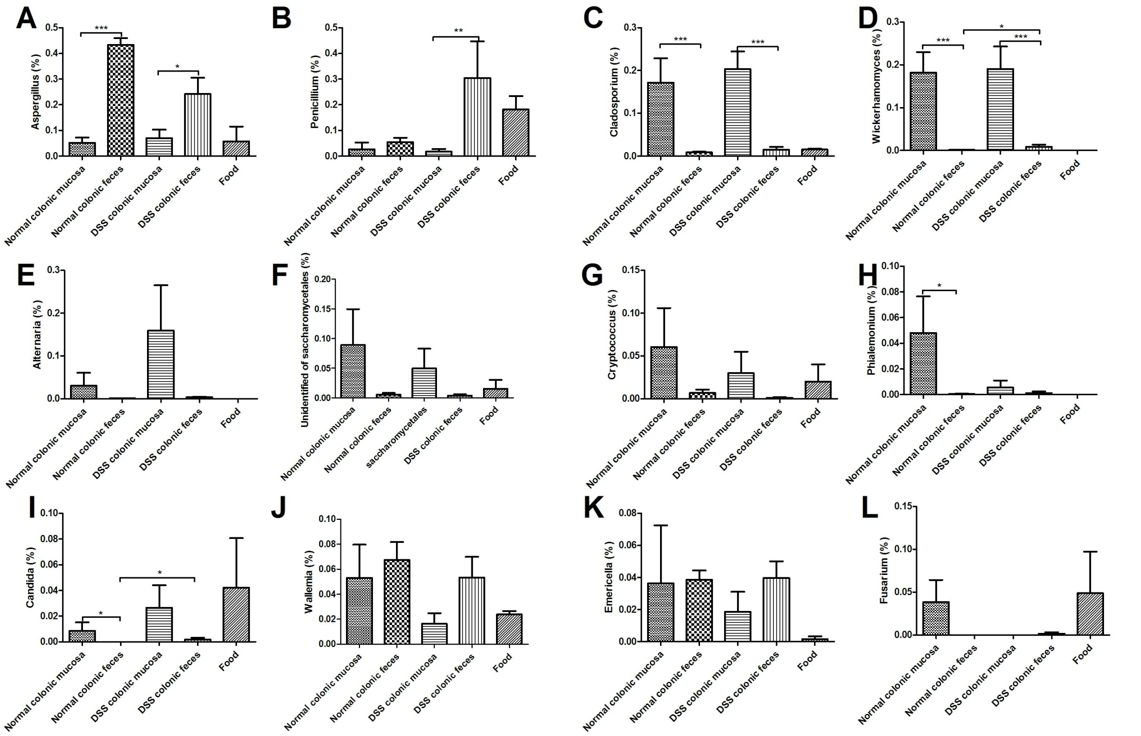

Supplementary Figure S4. Identification of the 12 major fungal microbiome at the genus level in the mouse food and in mucosal and fecal samples from mice with or without acute DSS-induced colitis. Samples from mice: n = 8; Samples from mouse food: n = 2. *$P < 0.05$, **$P < 0.01$, ***$P < 0.001$.

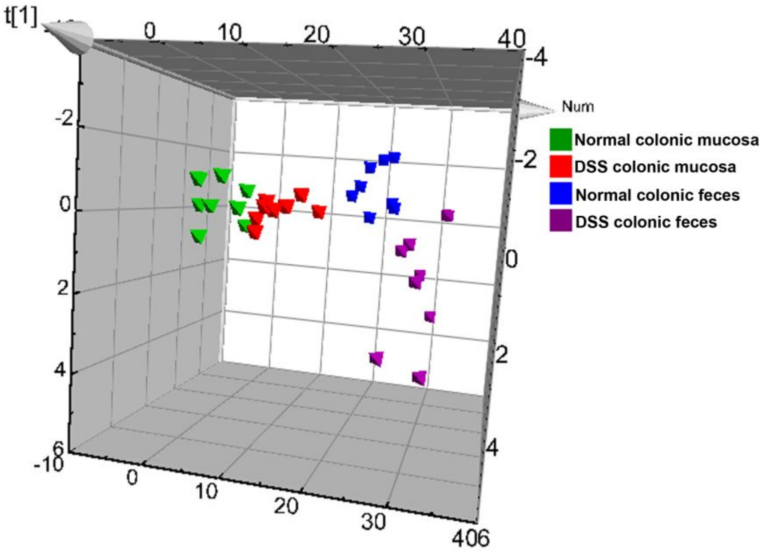

Supplementary Figure S5. Partial least-squares discriminant analysis (PLS-DA) scores plot shows that fungal compositions differ between the mucosa and the feces and change during intestinal inflammation. n = 8/group.

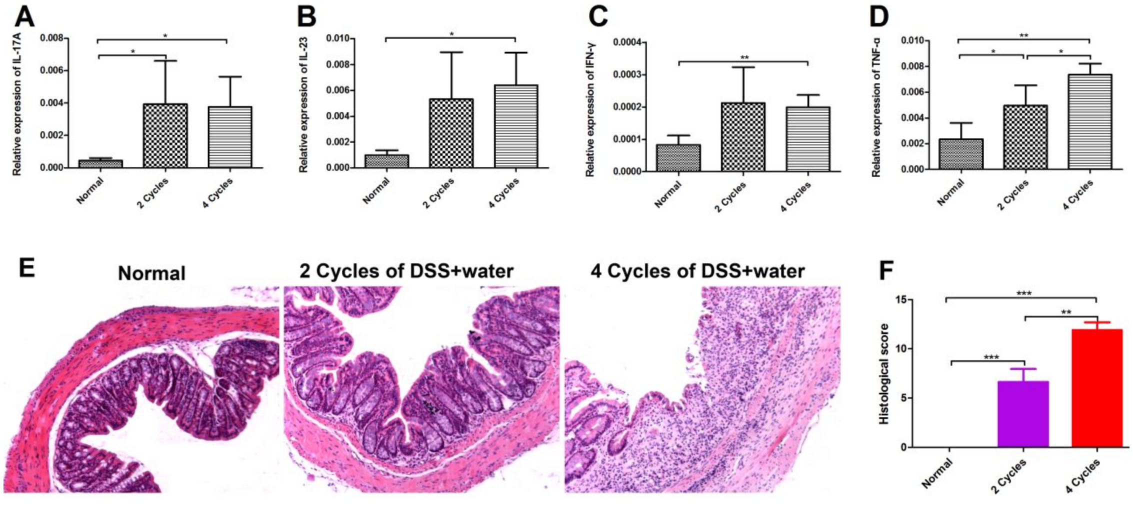

Supplementary Figure S6. Mice treated with four cycles of DSS+water exhibit more severe colonic inflammation than normal mice or mice treated with two cycles of DSS+water. (A–D) The mRNA levels of pro-inflammatory cytokines (IL-17A, IL-23, IFN-γ, and TNF-α) in the colonic mucosa from normal mice and mice treated with two and four cycles of DSS+water were detected by

real-time PCR. **(E–F)** Hematoxylin and eosin staining of representative cross-sections and the histological score of the murine distal colon (HE, ×200) (n = 8–12/group). *$P < 0.05$, **$P < 0.01$, ***$P < 0.001$.

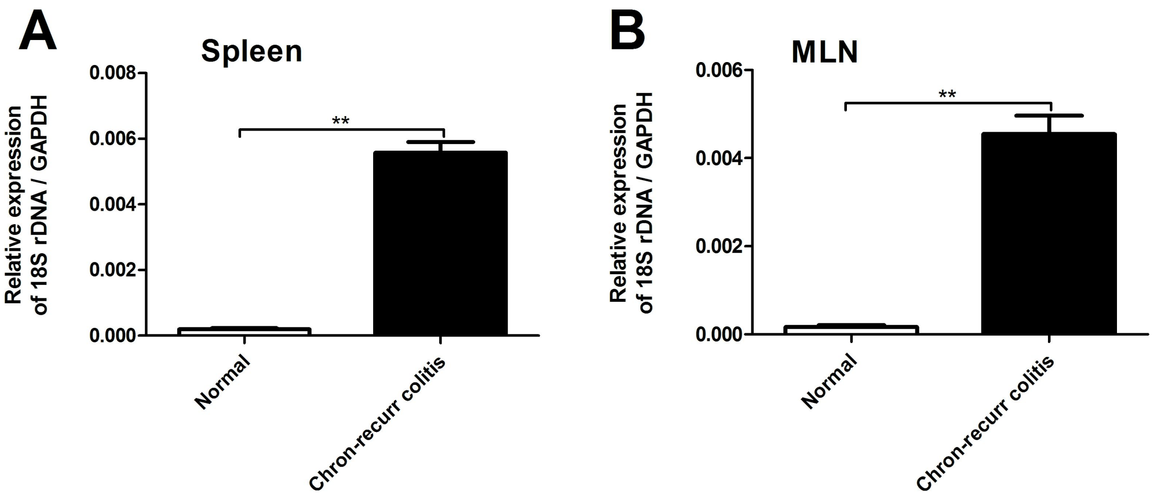

Supplementary Figure S7. Fungal translocation in mice with chronic recurrent (Chro-recurr) colitis. 18S rDNA level in spleen (**A**) and MLN (**B**) was significantly increased in mice with Chro-recurr colitis compare to the normal control. Chron-recurr colitis mice were treated with four 14-day cycles consisting of seven days of water containing 2.5% DSS followed by seven days of distilled water (DSS+water). 18S rDNA level was determined by qRT-PCR normalized to glyceraldehyde-3-phosphate dehydrogenase (GAPDH). (n=6-7 / group) *P < 0.05, **P < 0.01.

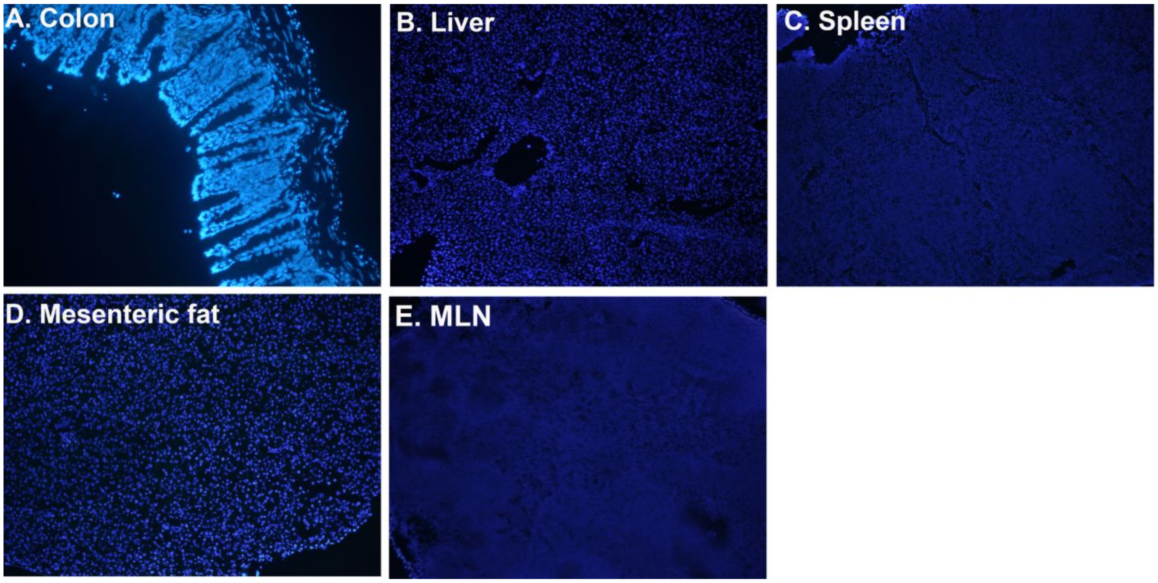

Supplementary Figure S8. (**A**) Colon, (**B**) liver, (**C**) spleen, (**D**) mesenteric fat and (**E**) mesenteric lymph node (MLN) sections from fluconazole treated mice (0.5 mg/ml for 23 days in the drinking water) were stained with anti-fungal antibody (green) and counterstained with DAPI (blue) and used as the control staining. (×400)

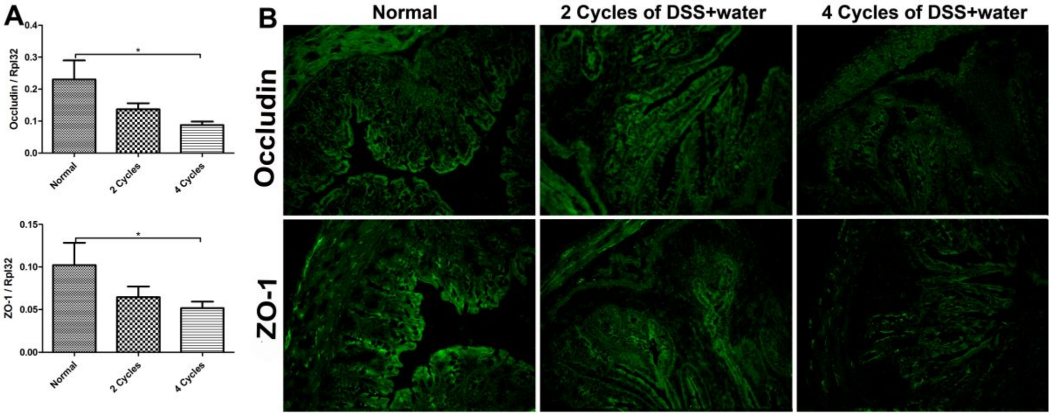

Supplementary Figure S9. Occludin and ZO-1 are decreased in mice with chronic recurrent colitis. (A) The mRNA expression levels of occludin and ZO-1 decreased in mice with two and four cycles of DSS+water compared with normal controls, with greater decreases in mice with four cycles

of DSS+water. *$P < 0.05$. **(B)** Immunofluorescence localization of occludin and ZO-1 in mouse distal colon showed morphological changes similar to those observed for mRNA levels in the colon. (×400)
